# Tensile Strength Statistics of High-Performance Mono- and Multifilament Polymeric Materials: On the Validity of Normality

**DOI:** 10.3390/polym15112529

**Published:** 2023-05-31

**Authors:** Yuri M. Boiko, Vyacheslav A. Marikhin, Liubov P. Myasnikova

**Affiliations:** Laboratory of Physics of Strength, Ioffe Institute, 26 Politekhnicheskaya Street, St. Petersburg 194021, Russia; v.marikhin@mail.ioffe.ru (V.A.M.); liu2000@mail.ru (L.P.M.)

**Keywords:** oriented polymers, strength, statistics, normality tests

## Abstract

Recently, the statistical distributions of the mechanical properties, including tensile strength (*σ*), of several high-strength high-modulus oriented polymeric materials have been analyzed by employing the Weibull’s and Gaussian statistical models. However, a more detailed comprehensive analysis of the distributions of the mechanical properties of these materials aimed to estimate the validity of normality by employing some other statistical approaches, is needed. In the present work, the *σ* statistical distributions of the seven high-strength oriented polymeric materials based on the polymers with three different chain architectures and conformations, ultra-high-molecular-weight polyethylene (UHMWPE), polyamide 6 (PA 6), and polypropylene (PP), each in the form of both single and multifilament fibers, have been investigated using graphical methods, such as the normal probability and quantile–quantile plots, and six selected formal normality tests, such as the Kolmogorov–Smirnov, Shapiro–Wilk, Lilliefors, Anderson–Darling, D’Agostino–K squared, and Chen–Shapiro tests. It has been found that the conformity of the *σ* distribution curves to the normal distribution, including the linearity of the normal probability plots, for the materials with lower strengths (*σ* < 1 GPa, quasi-ductile PA 6- and PP-based materials) is more correct as compared to those for the materials with markedly higher strengths (*σ* > 4 GPa, quasi-brittle UHMWPE-based materials). The impact of the sample type (single or multifilament fibers) on this behavior turned out to be negligible.

## 1. Introduction

Currently, owing to the elaboration of the structural-kinetic approach of the orientational strengthening of polymers [1], it was possible to produce high-strength high-modulus materials with extremely high values of tensile strength (up to *σ* = 6 GPa) and Young’s modulus (up to *E* = 230 GPa) [2], which are comparable with their theoretical estimates and markedly higher, by two orders of magnitude, than those of non-oriented polymers. In addition, their long-term mechanical properties, such as stress relaxation are satisfactory [3]. These polymer materials are very perspective when used as reinforcing components of high-performance composite materials since their specific (per unit of weight) mechanical properties are markedly higher as compared to those of high-performance inorganic materials [4,5]. To ensure the high reliability of operation of these materials, it is necessary to develop physically substantiated and correct methods for determining the statistically proper values of mechanical properties. Undoubtedly, these methods can be based on the analysis of only a large test number, tens or hundreds of measurements of identical samples [2,4,5,6,7,8,9,10,11,12,13,14,15,16,17], rather than the widely used three to five samples only [18,19,20,21]. Despite a marked increase in labor intensity in the former case, this approach allows one not only to estimate the average value of mechanical characteristics with a high degree of reliability, but also to obtain valuable information about the physical nature of their statistical distributions. This additional information is useful for a better understanding of the deformation and fracture mechanisms of high-performance materials. For instance, if the strength distribution conforms to the standard Weibull’s distribution function [2,4,5,6,7,8,9,10,11,12,13,14,15,16,17], it means that the fracture mechanism is controlled by surface or interface cracks [2,6,22,23]. In contrast, if the Gaussian model is valid while the Weibull’s one is not, it implies that this process is controlled by the sum of many independent and equally-weighed factors [8,24], i.e., it is a random process. For instance, it is expected that the material’s brittleness should have an impact on the type of statistical distribution of a mechanical property. In particular, this factor becomes critical for brittle and quasi-brittle materials to which the high-performance polymer materials belong, and for which the data scatter is rather broad [2,4,5,6,7,8,9,10,11,12,13,14,15,16,17,22,23]. 

Recently, we used this approach [25] to analyze the statistical distributions of *σ* of a number of high-strength high-modulus oriented polymeric materials by employing both the Weibull’s (the critical nature of the occurrence of localized processes on the surface) and Gaussian (the equal probability nature of the processes in the volume) statistical models. It has been shown that both the chain architecture (ultra-high-molecular-weight polyethylene (UHMWPE), polyamide 6 (PA 6), or polypropylene (PP)) and the sample type (thin single film threads and fibers, on the one hand, and multifilament fibers consisting of some hundreds of individual fibers, on the other hand) impact both on the most correct type (Weibull’s or Gaussian) of the *σ* distributions and their statistical parameters [the Weibull’s modulus and the standard deviation of the mean (SD)]. For instance, the Weibull’s model was more correct than the Gaussian one for the quasi-brittle ultra-high-strength UHMWPE-based materials (*σ* up to 6 GPa), while both of these models were appropriate for the ductile PA 6- and PP-based materials characterized with the *σ* values by a lower order of magnitude. However, a broader look at the most widely used statistical distribution, the normal distribution, by involving a number of other available methods [26,27,28,29,30,31,32,33,34,35,36,37], has not yet been considered to analyze the distributions of the mechanical properties of these materials, though it could be helpful for revealing further details in their normal distributions. 

Naturally, assessing the assumption of normality is required by most statistical procedures in order to prove its validity. The easiest and effective diagnostic tool for checking the normality of data is to use graphical methods including the normal probability and quantile–quantile plots, and the histograms of the probability density function (PDF) over the entire range of a property (e.g., of *σ*) variation. However, the use of graphical methods only is not sufficient. To further prove the validity of normality obtained in the graphical methods, formal normality tests should also be performed. A number of the proposed normality tests are very large (e.g., 50 have been reported in Reference [27]). However, the most often used are the Kolmogorov–Smirnov, Shapiro–Wilk, Lilliefors, Anderson–Darling, D’Agostino–K squared, and Chen–Shapiro tests [27,28,29,30,31,32,33,34,35,36,37]. For this reason, these six tests have been employed in the present work for checking the normality of seven data sets each of which included 50 fracture tests of identical samples. 

All the normality tests listed above are based on the idea of the null hypothesis (*H*_0_), which can be rejected or accepted. *H*_0_ of these tests is that the population of a given data set is normally distributed. To test whether or not *H*_0_ is true, the so-called *p*-value (the parameter of normality) is used. It is defined as the probability under the assumption of no effect or no difference (*H*_0_) of obtaining a result equal to or more extreme than what was actually observed. The *p*-value is a probability parameter varying between 0 and 1, which is usually automatically calculated using statistical software employing different statistical equations. For this reason, the *p*-value estimated in one test is different from the *p*-value estimated in the other test. However, for all the normality tests used, the critical *p*-value is defined to be 0.05, which is generally accepted [26,27,28,29,30,31,32,33,34,35,36,37]. This means that the null hypothesis is rejected when the *p*-value is smaller than 0.05 and accepted when the *p*-value is equal to or larger than 0.05. Therefore, *p*-values calculated in various tests for normality will be, first, analyzed with respect to the critical “cut-off” *p*-value = 0.05, and, second, compared with each other and with 1 (the strongest evidence for accepting *H*_0_), taking into account that the smaller the *p*-value, the stronger the evidence for rejecting *H*_0_.

Let us briefly mention some differences between the various normality tests employed [27,28,29,30,31,32,33,34,35,36,37]. The Kolmogorov–Smirnov test quantifies a distance between the empirical distribution function of the “sample” (i.e., the data set) and the cumulative distribution function of the reference distribution. The Lilliefors test is its improved modification. The D’Agostino–K squared test is an extension of the Shapiro–Wilk test. Along with its omnibus option, it has two more additional options, skewness and kurtosis, which characterize the lack of symmetry and whether the data are heavy-tailed or light-tailed relative to a normal distribution, respectively. The Shapiro–Wilk test examines *H*_0_ as to whether or not a data set came from a normally distributed population. The Anderson–Darling test originates from the Kolmogorov and Smirnov test, which is more powerful than the latter. In its basic form, it assumes that there are no parameters to be estimated in the tested distribution, in which case the test and its set of critical values are distribution-free. The Chen–Shapiro test compares the spacings between order statistics with the spacings between their expected values under normality, and is among the best choice for symmetric distributions and when the nature of non-normality is unknown. The use of these tests makes it possible to detail the mechanisms of deformation and fracture by considering various types of deviations from the classical Gaussian function.

Therefore, the goal of this work is to carry out a detailed comprehensive statistical investigation on the *σ* distributions of several high-strength polymer materials of different chain architectures (UHMWPE, PA 6, and PP) and sample types (mono- or multifilament fibers) using, for the first time, (i) the graphical methods including the normal probability and quantile–quantile plots, and (ii) a number of the formal normality tests of common use listed above in order to elucidate which of these statistical approaches is most appropriate. For this purpose, the oriented materials produced from the three polymers, UHMWPE, PA 6, and PP were selected. The choice of these polymers was motivated, first, by their different chain architectures and conformations resulting in their different drawability and, therefore, different attainable strengthening. Second, each of these polymers has been investigated in the two different sample types: (i) Single thin film threads or fibers and (ii) multifilament fibers. The importance of introducing this factor (mono- or multifilament) follows from the different statistical natures of these two types of samples. It consists of the “non-statistical origin” of a single fiber, on the one hand, and in the “statistical origin” of a multifilament sample representing some hundreds of individual fibers, on the other hand.

## 2. Materials and Methods

**Figure 1:**
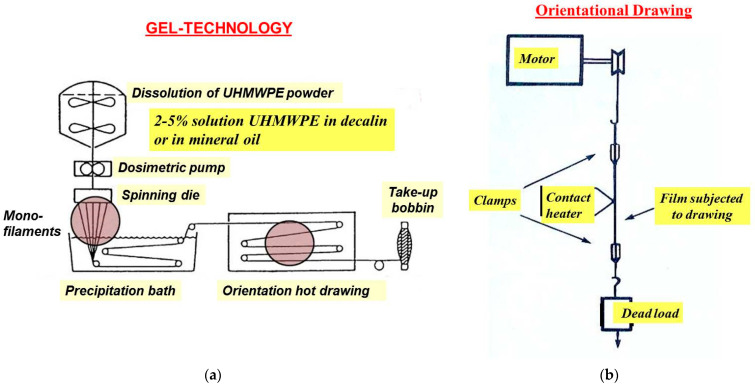
Fabrication of high-strength UHMWPE fibers (**a**) by gel-technology in industry and (**b**) by multi-stage hot-zone drawing technique using a home-built laboratory device.

The following samples were chosen for this investigation: Lab-scale single UHMWPE film threads ultradrawn to a very high draw ratio of 120 cast (i) from decalin or (ii) from virgin mineral oil kindly supplied by Honeywell, Charlotte, NC, USA, (iii) commercial gel-spun (gel-technology is schematically depicted in Figure 1a) multifilament fibers of UHMWPE (Dyneema SK60, Heerlen, the Netherlands) consisting of some hundreds of single fibers kindly supplied by *DSM*, the Netherlands, and commercially-available oriented single and multifilament fibers of (iv, v) PA 6 and (vi, vii) PP. In total, seven sample series have been investigated. The lab-scale oriented single film threads were produced from an UHMWPE nascent powder with a viscosity-average molecular weight of 3000 kg/mol synthesized at Boreskov Institute of Catalysis (Novosibirsk, Russia). A 1.5-wt% UHMWPE solution in decalin or in vaseline oil was prepared at a temperature of 160 °C under stabilization of 0.5 wt% of an antioxidant (di-tert-butyl-p-cresol). The UHMWPE gel films were produced by casting a polymer dilute solution into a Petri dish followed by quenching at ambient temperature. After drying, the gel was transformed into a xerogel in the form of films of a thickness of 0.1 mm. The as-produced xerogel films were cut into strips of a width of 1 mm, which were subjected to a multi-stage hot-zone drawing on a home-built device equipped with a pin heater [1] (see Figure 1b). The choice of two solvents was motivated by some differences in mechanical properties for commercial gel-spun UHMWPE fibers cast from decalin (Dyneema fibers, *DSM*, Heerlen, the Netherlands) and from virgin mineral oil (Spectra fibers, Honeywell, Charlotte, NC, USA). In the present work, the use of these two solvents was aimed to investigate a possible impact of the solvent type on the applicability of the normal distribution to the strength statistical behavior. The commercial single and multifilament fibers of UHMWPE, PA 6, and PP were used as received.

The samples were subjected to tensile loading on an Instron-1122 tensile tester (Norwood, Norwood, MA, USA) at ambient temperature at a cross-head speed of 10 mm/min with an initial distance between the tester clamps of 10 mm for the lab-scale UHMWPE single film threads (of a width of 0.4 mm and a thickness of ~0.002 mm), and at a strain rate of 200 mm/min and a distance between the tester clamps of 500 mm for all the five commercial materials: (i) Multifilament fibers of UHMWPE (linear density *D* = 179 tex), (ii) single filament with a diameter *d* of 0.20 mm and (iii) multifilament fibers (*D* = 213 tex) of PA 6, and (iv) single fiber (*d* = 0.17 mm) and (v) multifilament fibers of PP (*D* = 1087 tex). To estimate the statistically proper values of the tensile strength, 50 identical samples of each of the above-listed materials were tested.

The statistical distributions of the tensile strengths of the chosen high-performance polymer materials have been investigated using a number of the normality tests, such as the Kolmogorov–Smirnov, Shapiro–Wilk, Lilliefors, Anderson–Darling, D’Agostino–K squared, and Chen–Shapiro tests, and the normal probability and quantile–quantile plots. When constructing the two latter tests, one obtains the same results—quantitatively, the arithmetic average value (*σ*_av_) and the standard deviation of the mean (SD), and, qualitatively, a visual criterion of the curve linearity.

## 3. Results and Discussion

**Figure 2:**
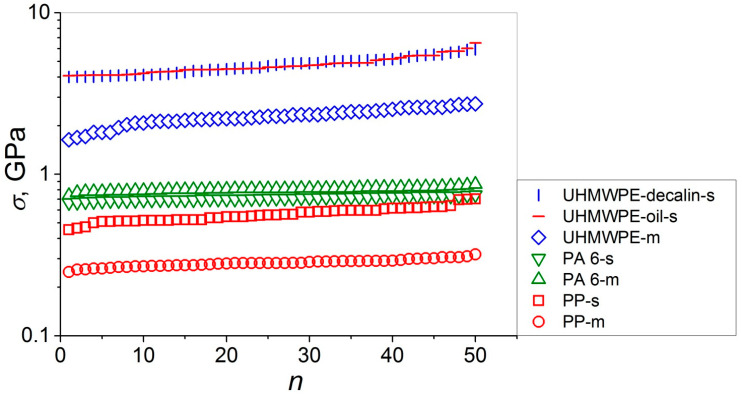
Semi-log plots of tensile strength *σ* as a function of sample number *n* in ascending order for single (-s) and multifilament (-m) film threads and fibers of UHMWPE, PA 6, and PP.

**Figure 3:**
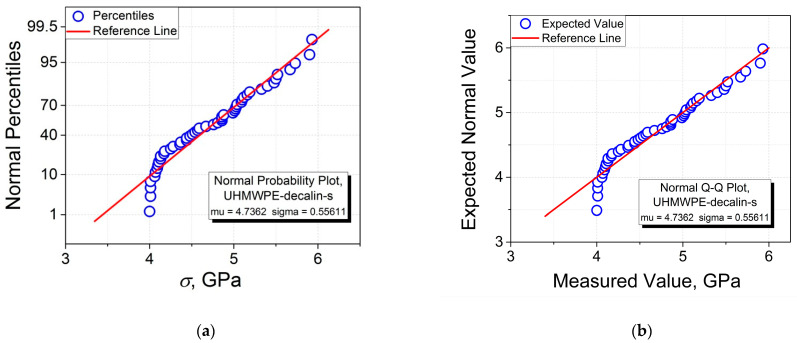

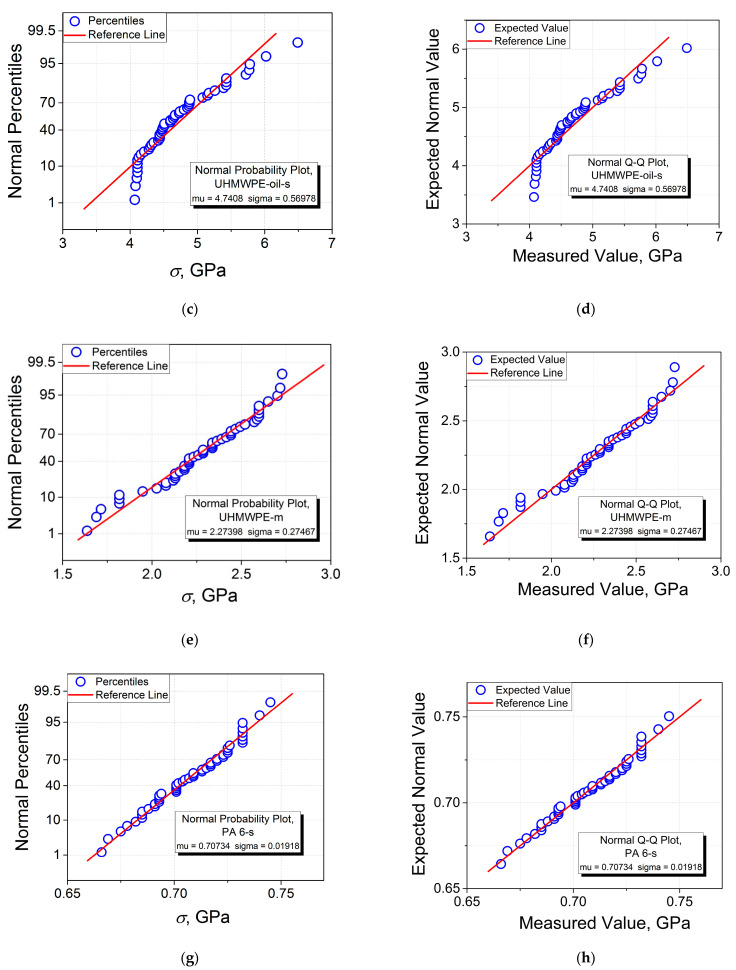

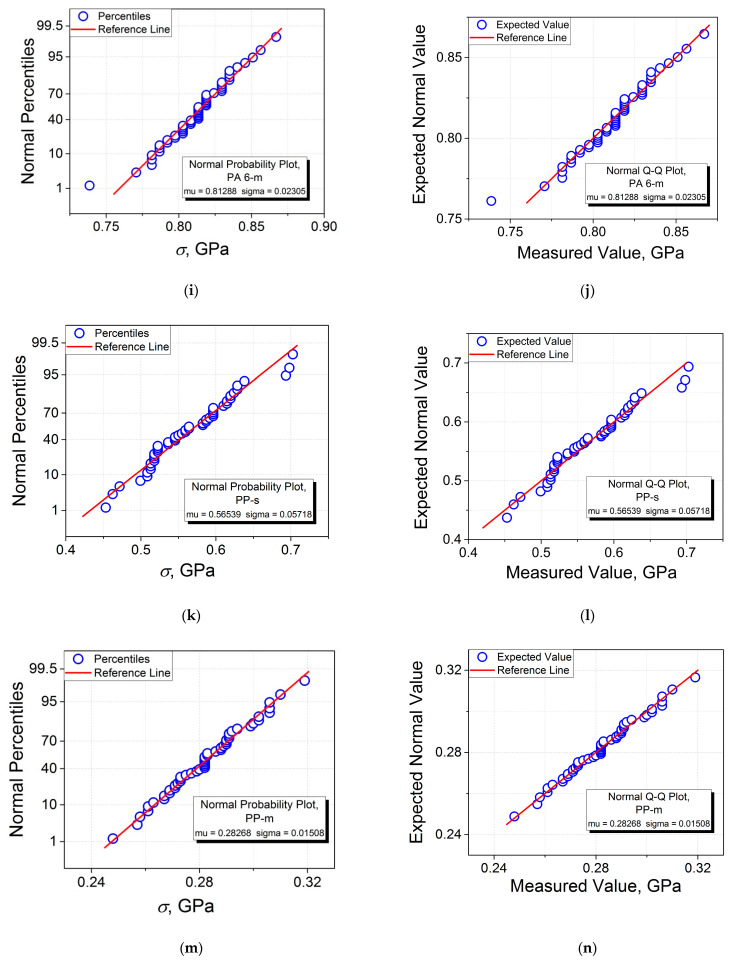
Normal probability and quantile–quantile plots of tensile strength *σ* for single (s) and multifilament (m) fibers of UHMWPE, PA 6, and PP: Single UHMWPE fibers cast from (**a**,**b**) decalin and (**c**,**d**) paraffin oil, (**e**,**f**) UHMWPE multifilament fibers, (**g**,**h**) PA 6 single fibers, (**i**,**j**) PA 6 multifilament fibers, (**k**,**l**) PP single fibers, and (**m**,**n**) PP multifilament fibers.

The data sets of the measured values of the tensile strength of the high-performance single and multifilament fibers of PP, PA 6, and UHMWPE arranged in the ascending order are reported in Figure 2. These seven data sets have been analyzed using OriginLab statistics software. First, the normal probability plots (see Figure 3a,c,e,g,i,k,m) and the corresponding quantile–quantile plots (see Figure 3b,d,f,h,j,l,n) were constructed. Thereafter, the six tests for normality have been performed for each of the materials. The results obtained from these tests are collected in Table 1 and Table 2.

Comparing the results obtained when constructing the normal probability plots with those of the corresponding quantile–quantile plots, it is seen that, as expected, the two graphical procedures give one and the same values of “mu” (*σ*_av_) and “sigma” (SD) for all the samples investigated. For instance, for the PP-m sample, one obtains *σ*_av_ = 0.28268 GPa and SD = 0.01508 GPa (see Figure 3m,n). Therefore, any of these plots can be chosen for a comparative analysis including the estimation of *σ*_av_ and SD.

As follows from the normal probability and quantile–quantile plots, qualitatively, they have a linear shape for the samples of UHMWPE-m (see Figure 3e,f), PA 6-s (see Figure 3g,h), PA 6-m (see Figure 3i,j), PP-s (see Figure 3k,l), and PP-m (see Figure 3m,n), while those for the samples of UHMWPE-d-s (see Figure 3a,b) and UHMWPE-o-s (see Figure 3c,d) demonstrate the deviation from linearity at smaller strengths. Therefore, it may be concluded from the graphical analysis performed that the normal distribution seems not to be appropriate in the two latter cases.

Let us introduce a parameter characterizing the data scatter (SD) normalized by *σ*_av_, the ratio of “sigma” (SD) to the corresponding values of “mu” (*σ*_av_), SD/*σ*_av_, expressed in percentage as (SD/*σ*_av_) × 100%. The largest values of SD/*σ*_av_ estimated from this analysis, which are close, were obtained for the three UHMWPE samples: UHMWPE-d-s (SD/*σ*_av_ = 11.7%), UHMWPE-o-s (SD/*σ*_av_ = 12.0%), and UHMWPE-m (SD/*σ*_av_ = 12.1%). The smallest and intermediate SD/*σ*_av_ values were calculated for PA 6-s (SD/*σ*_av_ = 2.7%) and PA 6-m (SD/*σ*_av_ = 2.8%), and for PP-s (SD/*σ*_av_ = 10.1%) and PP-m (SD/*σ*_av_ = 5.3%), respectively. These results indicate that the data scatter is broader for the UHMWPE-based materials characterized by markedly higher (up to by an order of magnitude—see Figure 2) strengths, on the one hand, and by smaller engineering strains at break *ε*_b_ <5% as compared to *ε*_b_ = 10% and *ε*_b_ = 16–18% for the PA 6- and PP-based materials, respectively [25], on the other hand. This behavior seems to be reasonable since the former ones are quasi-brittle (the role of an occasional sample fracture increases), while the latter are ductile, which, preserving their brittle fracture, results in a decrease in the observed data scatter. The analysis performed has found that, in general, the sample type has the minor effect on the data scatter (SD) normalized by *σ*_av_. Actually, for the single and multifilament fibers of both UHMWPE and PA 6, the values of (SD/*σ*_av_) × 100% almost coincide; for UHMWPE: 11.7–12.0% (s) and 12.1% (m), respectively, and for PA 6: 2.7% (s) and 2.8% (m), respectively. In the case of the PP-based materials, a smaller value of (SD/*σ*_av_) × 100% of 5.3% has been estimated for the multifilament fibers as compared to those of 10.1% for the single fiber. This behavior may be explained by an extremely large number of the fractured individual fibers per one test for the multifilament sample including about two hundreds of single fibers with respect to the single fiber (i.e., at a ratio of 200 to 1), making the results for the former more proper statistically and giving a more reliable strength variation interval.

**Figure 4:**
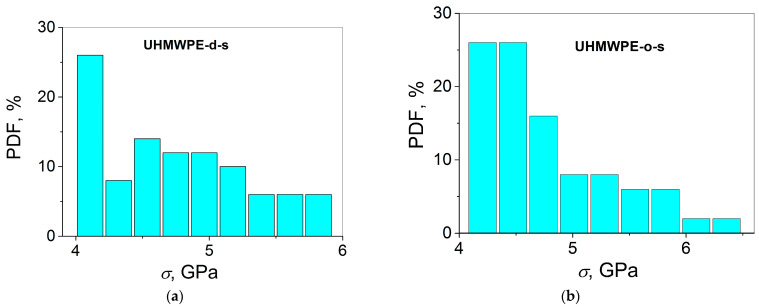

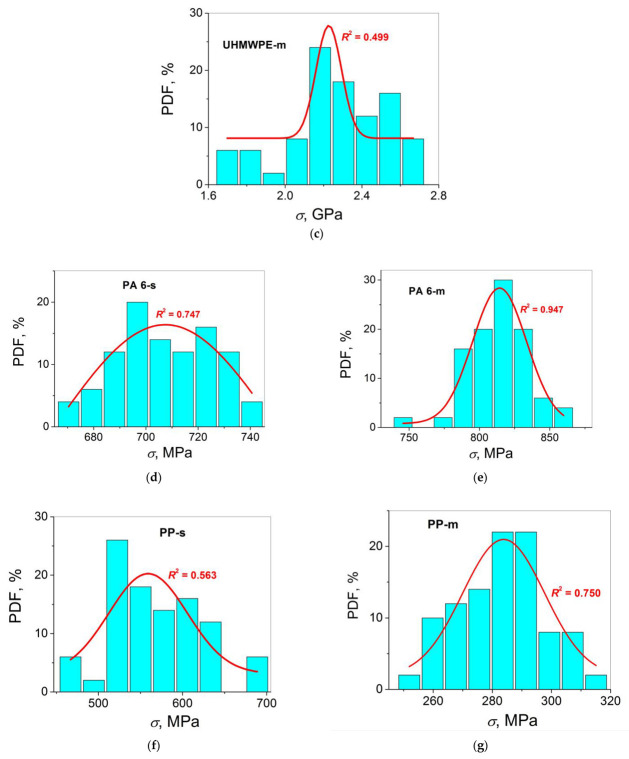
Histograms PDF(*σ*) for the UHMWPE single fibers cast from (**a**) decalin and (**b**) paraffin oil, (**c**) UHMWPE multifilament fibers, (**d**) PA 6 single fibers, (**e**) PA 6 multifilament fibers, (**f**) PP single fibers, and (**g**) PP multifilament fibers.

Let us now turn to the results of the analysis of the tensile strength distributions of the seven data sets under investigation using several normality tests which are presented in Table 1 and Table 2. As follows from this analysis, almost all the tests for normality performed give a positive answer to the question concerning the expected validity of the normal distribution to describe correctly the *σ* distributions, except for the following cases for the samples of UHMWPE-d-s and UHMWPE-o-s: The Shapiro–Wilk and Anderson–Darling tests (both UHMWPE-d-s and UHMWPE-o-s), the Lilliefors and D’Agostino–K squared tests (only UHMWPE-o-s). In general, these results are in accordance with the histograms PDF(*σ*) for the corresponding samples (see Figure 4). Actually, one observes the bell Gaussian curves drawn through the histograms PDF(*σ*) only for the samples of UHMWPE-m, PA 6-s, PA 6-m, PP-s, and PP-m. In contrast, these curves could not be constructed for the samples of UHMWPE-d-s and UHMWPE-o-s, which agrees with the deviation from linearity at small strengths on the probability plots presented in Figure 3a–d. However, in some cases, there is a disagreement between the results of the normality tests presented in Table 1 and Table 2 and the histograms PDF(*σ*) shown in Figure 4. For instance, the best results describing the *σ* distributions using the Gaussian model (the observation of well-defined bell-shaped curves) have been received for the PA 6-m (see Figure 4e) and PP-m samples (see Figure 4g). Therefore, it is expected that the normality tests for the *σ* distributions of these two materials should also give similar (i.e., best fitting) results. To address this issue, let us compare the *p*-values from Table 1 and Table 2 estimated in the six normality tests for the seven high-strength materials. First, it should be noted that different tests have produced different *p*-values for one and the same data set since each of them uses different statistical equations (which are not presented here) employed for calculations. However, all the *p*-values were compared with the two critical *p*-values: 0.05 and 1. As mentioned above, on the one hand, the test rejects the null hypothesis of normality when the p-value is less than or equal to 0.05. Therefore, the majority of the p-values collected in Table 1 which are larger than 0.05 meet this requirement, except for some of those estimated in the Shapiro–Wilk and Anderson–Darling tests (UHMWPE-d-s and UHMWPE-o-s), and in the D’Agostino–K squared test (UHMWPE-o-s).

Probably, the exceptions found indicate that those tests are most appropriate for the investigated samples since the UHMWPE-d-s and UHMWPE-o-s samples, especially the latter, demonstrate the skewed distributions (see Figure 4a,b) which are not reminiscent of the classical Gaussian bell curve.

On the other hand, the best fitting result is obtained when the *p*-value is larger, especially when it attains 1. From this point of view, the Kolmogorov–Smirnov test seems to be more appropriate since the six *p*-values of the total seven calculated (i.e., 86%) are larger than the (rather high) arbitrary chosen *p*-value of 0.5, among which the three *p*-values are higher than 0.87, attaining even 1. Thereafter, the D’Agostino–K squared test follows for which the six *p*-values of the total twenty one calculated (i.e., 29%) are larger than 0.5. For the three other tests, the Shapiro–Wilk, Lilliefors, Anderson–Darling tests, the results are less satisfactory: One, zero, and one *p*-value > 0.5, respectively, per seven calculated *p*-values in total, i.e., 14% (PP-m), 0% (no *p*-values > 0.5), and 14% (PP-m), respectively. Moreover, it should be noted that the best correlations between the observation of the well-defined bell curve and the highest *p*-value have been found only for one sample, the PP-m sample: The *p*-value = 0.9436 (the Shapiro–Wilk test), *p*-value = 0.7512 (the Anderson–Darling test), and *p*-values = 0.96313, 0.79097, and 0.94429 (the D’Agostino–K squared tests). For the PA 6-m sample, for which the best Gaussian fitting result is obtained with the highest root mean square deviation *R*^2^ = 0.947, the *p*-value is always smaller not only than the corresponding *p*-value for the PP-m sample for which the fitting result with *R*^2^ = 0.750 is less satisfactory, but also than the corresponding *p*-values for other materials investigated (UHMWPE-d-s and UHMWPE-m in the Kolmogorov–Smirnov test, PA 6-s in the Shapiro–Wilk test, UHMWPE-o-s in the Lilliefors test, PA 6-s in the Anderson–Darling test, PP-s, PA 6-s, UHMWPE-m, and UHMWPE-d-s in the D’Agostino–K squared test) for which the bell curves could not be constructed correctly. Therefore, the positive answers received from the normality tests (“cannot reject normality”) do not guarantee that the corresponding histograms in the coordinates PDF(*σ*) will represent the bell curves which are typical for the normal distribution. Nevertheless, principally, the Shapiro–Wilk and Anderson–Darling tests seem to be more correct since the result “reject normality” obtained for the UHMWPE single film threads only in these two tests coincide with the shape of the distribution histograms (no bell curves are observed) for one and the same samples.

Therefore, all the tests applied for normality are not straightforward to conclude the validity of the normal distribution.

Moreover, when comparing the normal probability and quantile–quantile plots shown in Figure 3 with the corresponding histograms PDF(*σ*) shown in Figure 4, one may notice the differences observed in the distribution behaviors. First, qualitatively, the most correct linearity of the normal probability and quantile–quantile plots has been observed for the four samples: PA 6-s (see Figure 3g,h), PA 6-m (see Figure 3i,j), PP-s (see Figure 3k,l), and PP-m (see Figure 3m,n).

As a lower limit of the conformity of the strength variation to the Gaussian distribution can be chosen, the PDF(*σ*) histograms fitted with a root mean square reliability of *R*^2^ = 0.5, when the bell curve is still observed (UHMWPE-m, see Figure 4c). In this way, only the PDF(*σ*) histograms for the UHMWPE-d-s and UHMWPE-o-s samples do not conform to the Gaussian distributions, as observed in Figure 4a,b. Therefore, at first glance, it is expected that the *p*-values for the UHMWPE-m sample can also be considered as the limits of the validity of normality in the frameworks of the normality tests performed. To put it differently, in this case, the *p*-value for the UHMWPE-m sample estimated in each of the normality tests should be higher as compared to those for the UHMWPE-d-s and UHMWPE-o-s samples, but lower as compared to those for the PA 6-s, PA 6-m, PP-s, and PP-m samples. However, contrary to expectations, this assumption is valid for the Shapiro–Wilk test only, but not valid for all the other tests (see Table 1). Moreover, these discrepancies can be found for the PA 6-s (*R*^2^ = 0.747) and the PA 6-m samples (*R*^2^ = 0.947) since one obtains larger *p*-values for the PA 6-s sample as compared to those for the PA 6-m sample in the Kolmogorov–Smirnov, Shapiro–Wilk, Lilliefors, Anderson–Darling, and three D’Agostino–K squared tests. Nevertheless, one observes a full correlation between the *R*^2^ values and the *p*-values for the PP-based samples. Indeed, a larger *R*^2^ value of 0.750 for the PP-m sample as compared to those of 0.563 for the PP-s sample correlates with larger *p*-values for the former as compared to those for the latter in all the normality tests employed.

As far as the solvent type used for casting the UHMWPE gel single film threads is concerned, one can observe the influence of this factor on the results of the tests for normality. The principal difference is revealed in the following two tests—the Lilliefors and D’Agostino–K squared tests, which consists of receiving the results “cannot reject normality” and “reject normality” for the UHMWPE-d-s and UHMWPE-o-s samples, respectively. These results correlate with a more correct linearity of the normal probability plot for the UHMWPE-d-s sample (see Figure 3a) as compared to the UHMWPE-o-s sample (see Figure 3c).

In the Chen–Shapiro test, the same 5% (0.02964) and 10% critical values (0.00336) were calculated for all the seven oriented samples analyzed. The only differences have been observed for the test statistic parameter which can be positive or negative. Based on these results, the evidence for accepting *H*_0_ (“cannot reject normality”) takes place only for the negative values of this parameter.

Therefore, it may be concluded that the results of the analysis of the conformity of the strength distribution curves to the normal distribution using the formal normality tests, in combination with the graphical method probing that receives the bell curves obtained by Gaussian fitting of the histograms PDF(*σ*) for high-strength polymer materials, have a complicated and in some cases contradictory character. To put it differently, the variation in the *R*^2^ values obtained in the Gaussian fitting did not correlate with the variation in the *p*-values obtained in all the normality tests applied. Therefore, the combined use of the graphical methods, such as the normal probability or quantile–quantile plots, and the histograms PDF(*σ*) of the formal normality tests and the Weibull’s analysis is recommended for the comprehensive statistical characterization of the mechanical properties of polymer materials concerning the conformity of the data distribution to normality. Only in this case, a detailed statistical picture of the mechanical behavior of the materials of various natures can be received. Nevertheless, among the tests for normality applied to the analysis of the tensile strength distributions of the seven high-strength polymeric materials, the following tests turned out to be more appropriate: From the point of view of the highest *p*-values—the Kolmogorov–Smirnov test; from the point of view of the best correlation with the Gaussian fitting—the Shapiro–Wilk and Anderson–Darling tests.

Finally, the observations of the bell curves which correspond better to the normal distribution for the multifilament samples of the three polymers with different chain architectures (see Figure 4c,e,g) as compared to those for the corresponding single fibers (see Figure 4a,b,d,f) confirm the hypothesis suggested in the Introduction about the different statistical natures of these two types of samples. Actually, the multifilament fibers possess more probability to demonstrate the normal distribution behavior since the probability for one fiber to break is averaged between many individual fibers (some hundreds). To put it differently, this process has a random statistical origin. In contrast, the critical role of surface cracks dominates in the fracture of a quasi-brittle single fiber, which corresponds better to the Weibull’s, not the random-like, normal, distribution [8].

## 4. Conclusions

For the first time, the comprehensive statistical analysis of the tensile strength distributions of the seven high-performance polymer materials differing in the chain architecture (carbon chain UHMWPE and PP, and carbon chain containing peptide bonds PA 6) and in the sample type (single or multifilament fibers) has been carried out using the graphical methods, such as the normal probability and quantile–quantile plots, and a number of the formal normality tests, such as the Kolmogorov–Smirnov, Shapiro–Wilk, Lilliefors, Anderson–Darling, D’Agostino–K squared, and Chen–Shapiro tests. It has been shown that the conformity of the *σ* distribution curves to the normal distribution, including the observation of linearity of the normal probability plots, is more correct for the materials with lower strengths (quasi-ductile PA 6 and PP, *σ* < 1 GPa) as compared to those with higher strengths (quasi-brittle UHMWPE, *σ* = 2–6 GPa). The best evidence to accept the null hypothesis due to attaining the highest possible *p*-value of 1 has been received in the Kolmogorov–Smirnov test, indicating that this test is more favorable for this class of materials. However, from the point of view of the best correlation with the Gaussian fitting results, the Shapiro–Wilk and Anderson–Darling tests have been shown to be more correct.

The influence of the sample type (single or multifilament fibers) on the data scatter, as characterized by the reduced statistical parameter SD/*σ*_av_, turned out to be negligible for each of the three polymers involved. In contrast, the chain architecture has been found to influence this parameter. In particular, the carbon chain UHMWPE and PP have demonstrated a broader strength distribution with respect to the carbon chain PA 6 with the inclusion of the peptide group in the chain backbone. Among the two carbon chain polymers investigated, the broadest data scatter (the highest SD/*σ*_av_ values) was observed for UHMWPE with in-plane trans-zigzag chain conformation as compared to those for PP with a spiral-like chain conformation.

It has been shown that the positive answers “cannot reject normality” received in the normality tests of the strength distribution curves do not guarantee that the corresponding fitting curves of the histograms PDF(*σ*) will represent the bell curves, which are characteristic of the Gaussian distribution. For the comprehensive statistical characterization of the mechanical properties of high-strength polymer materials, the complimentary use of the various widely used statistical approaches that we discussed in the present work and earlier should be involved.

## Figures and Tables

**Table 1:** Results of normality tests for the strength distribution of oriented single (s) and multifilament (m) fibers and film threads of UHMWPE, PA 6, and PP.

Sample	Test Type	Statistic	*p*-Value	Decision at Level 5% *
UHMWPE-d-s	Kolmogorov–Smirnov	0.10138	0.67024	+
UHMWPE-o-s		0.13729	0.2772	+
UHMWPE-m		0.08753	0.87868	+
PA 6-s		0.07667	1	+
PA 6-m		0.11073	0.5462	+
PP-s		0.11388	0.50771	+
PP-m		0.08202	0.96825	+
UHMWPE-d-s	Shapiro–Wilk	0.9401	0.01359	−
UHMWPE-o-s		0.90444	0.00068	−
UHMWPE-m		0.963	0.11868	+
PA 6-s		0.97839	0.48625	+
PA 6-m		0.97385	0.32946	+
PP-s		0.96401	0.13084	+
PP-m		0.98989	0.9436	+
UHMWPE-d-s	Lilliefors	0.10138	0.2	+
UHMWPE-o-s		0.13729	0.01947	−
UHMWPE-m		0.0875	0.2	+
PA 6-s		0.07667	0.2	+
PA 6-m		0.11073	0.1316	+
PP-s		0.11388	0.10418	+
PP-m		0.08202	0.2	+
UHMWPE-d-s	Anderson–Darling	0.79785	0.0361	−
UHMWPE-o-s		1.42074	0.001	−
UHMWPE-m		0.47891	0.22519	+
PA 6-s		0.35124	0.45618	+
PA 6-m		0.43822	0.28343	+
PP-s		0.59274	0.11797	+
PP-m		0.24411	0.7512	+
	D’Agostino–K squared:			
UHMWPE-d-s	Omnibus	4.58424	0.10105	+
	Skewness	1.21382	0.22482	+
	Kurtosis	−1.76377	0.07777	+
UHMWPE-o-s	Omnibus	9.79853	0.00745	−
	Skewness	2.89764	0.00376	−
	Kurtosis	1.18415	0.23635	+
UHMWPE-m	Omnibus	1.90622	0.38554	+
	Skewness	−1.37254	0.1699	+
	Kurtosis	−0.14954	0.88113	+
PA 6-s	Omnibus	2.04165	0.3603	+
	Skewness	−0.47715	0.63326	+
	Kurtosis	−1.34684	0.17803	+
PA 6-m	Omnibus	4.701	0.09532	+
	Skewness	−1.27661	0.20174	+
	Kurtosis	1.7525	0.07969	+
PP-s	Omnibus	1.71302	0.42464	+
	Skewness	1.28154	0.2	+
	Kurtosis	0.26585	0.79036	+
PP-m	Omnibus	0.07514	0.96313	+
	Skewness	0.26505	0.79097	+
	Kurtosis	−0.06988	0.94429	+

* “+” and “−” in column 5 mean “cannot reject normality” and “reject normality”, respectively.

**Table 2:** Statistical parameters estimated from the Chen–Shapiro normality test.

Sample	Statistic	10% Critical Value	5% Critical Value	Decision at Level 5% *
UHMWPE-d-s	0.0907	0.00336	0.02964	−
UHMWPE-o-s	0.21701	0.00336	0.02964	−
UHMWPE-m	−0.00231	0.00336	0.02964	+
PA 6-s	−0.05567	0.00336	0.02964	+
PA 6-m	−0.05105	0.00336	0.02964	+
PP-s	−0.0115	0.00336	0.02964	+
PP-m	−0.10142	0.00336	0.02964	+

* “+” and “−” in column 5 mean “cannot reject normality” and “reject normality”, respectively.

## Data Availability

Not applicable.
